# The Influence of Texting Language on Grammar and Executive Functions in Primary School Children

**DOI:** 10.1371/journal.pone.0152409

**Published:** 2016-03-31

**Authors:** Chantal N. van Dijk, Merel van Witteloostuijn, Nada Vasić, Sergey Avrutin, Elma Blom

**Affiliations:** 1 Department of Special Education, Cognitive and Motor Disabilities, Utrecht University, Utrecht, the Netherlands; 2 Department of Linguistics, University of Amsterdam, Amsterdam, the Netherlands; 3 Department of Language, Literature and Communication, Utrecht Institute of Linguistics, Utrecht University, Utrecht, the Netherlands; University of Akron, UNITED STATES

## Abstract

When sending text messages on their mobile phone to friends, children often use a special type of register, which is called *textese*. This register allows the omission of words and the use of *textisms*: instances of non-standard written language such as *4ever* (forever). Previous studies have shown that textese has a positive effect on children’s literacy abilities. In addition, it is possible that children’s grammar system is affected by textese as well, as grammar rules are often transgressed in this register. Therefore, the main aim of this study was to investigate whether the use of textese influences children’s grammar performance, and whether this effect is specific to grammar or language in general. Additionally, studies have not yet investigated the influence of textese on children’s cognitive abilities. Consequently, the secondary aim of this study was to find out whether textese affects children’s executive functions. To investigate this, 55 children between 10 and 13 years old were tested on a receptive vocabulary and grammar performance (sentence repetition) task and various tasks measuring executive functioning. In addition, text messages were elicited and the number of omissions and textisms in children’s messages were calculated. Regression analyses showed that omissions were a significant predictor of children’s grammar performance after various other variables were controlled for: the more words children omitted in their text messages, the better their performance on the grammar task. Although textisms correlated (marginally) significantly with vocabulary, grammar and selective attention scores and omissions marginally significantly with vocabulary scores, no other significant effects were obtained for measures of textese in the regression analyses: neither for the language outcomes, nor for the executive function tasks. Hence, our results show that textese is positively related to children’s grammar performance. On the other hand, use of textese does not affect—positively nor negatively—children’s executive functions.

## Introduction

With children’s increasing use of mobile phones, concerns have been raised about its influence on their literacy skills. One well-known feature of children’s text messages is that they do not always adhere to conventional written language rules and use a register that is called *textese*. In this register, children make use of phonetic replacements, such as *ur* instead of *your* and acronyms, such as *lol* [1] and drop words (e.g. [2]). This has led to the assumption that characteristics of textese may leak into children’s general writing, ultimately resulting in language deterioration [3,4]. However, this is in sharp contrast to findings from several studies showing that children who used textese frequently did not perform poorly on spelling and tasks measuring literacy abilities (see [5] for a review). More recently, this research has been expanded to the effect of textese on children’s grammar abilities in written language [2,6–8]. Outcomes of some studies suggest a negative influence of textese on grammar [7]. Nevertheless, variability in coding of textese between studies and use of written tasks, which do not strictly represent grammar, may have masked the effect of textese on children’s grammar abilities. Therefore, the main aim of the present study is to ascertain whether use of textese influences children’s grammar performance in spoken language.

Yet another understudied area is the connection between use of textese and children’s cognitive development. Previous studies have shown that young people who often switch between different media types and non-media (e.g. watching television while doing homework), have lower executive functions (see [9] for an overview). As many young children nowadays own a (smart)phone they may also be prone to this effect. On the other hand, children who are proficient in textese, might have similar advantages as bilingual children have, as they might be considered a special type of bilinguals—in a different modality—having to switch between formal written language and textese. This is so because various studies have shown superior performance on executive function tasks by bilingual children over monolingual children (see [10]). Thus, the second aim of this study is to determine whether proficient texters have better-developed executive functions than non-proficient texters, similar to proficient bilingual children.

### Textese and literacy

In the last decade, a number of studies have been conducted focusing on children’s text message writing and use of textese. Textese is a form of abbreviated written—or actually typed—language, that is characterized by the omission of words and the use of *textisms*, such as abbreviations, letter/number homophones, emoticons, etc. (see [5] for an overview of categories). Identified textism categories range from 4 [1] to 11 [11]. In public opinion, use of textese by children and young adults has been linked to poor reading and writing skills and even language deterioration, as illustrated by a corpus study by Thurlow [4], who investigated opinions on texting and textese in newspapers.

Some studies have indeed found negative associations between frequency of use of textese and measures of spelling [2] and other tasks measuring abilities related to literacy such as verbal and nonverbal reasoning [12]. However, in the majority of studies, children’s use of textese and their spelling and literacy abilities were found to be positively related: accuracy of reading textese and speed of reading and writing textese were positively associated with children’s spelling, reading and non-word reading scores [13]; and number of (certain types of) textisms and textism density—the ratio of textisms used per word—were positively associated with spelling skills [11,12,14–18]; orthographic processing ability [2]; phonological and phoneme processing, awareness and retrieval abilities [11,16–18]; verbal reasoning scores [12] reading skills [11,16–19]; and writing skills [17].

Nearly all studies on the effects of textese focused on children’s literacy development and hardly any attention has been paid to the effects of textese on children’s language development. Specifically grammatical development is interesting in this respect because, as mentioned by Kemp and colleagues [2], grammar rules of conventional written language are often transgressed in textese. As a result, this lack of grammatical conventions might leak into registers other than textese. To the best of our knowledge, only four studies have investigated effects of use of textese on children’s use of grammar [2,6–8]. These studies have focused on the use of grammar in written language and have shown mixed results. Cingel and Sundar [6] obtained a negative association between the number of text messages children sent and received and the number of textisms used and children’s grammar abilities. However, these findings are hard to interpret, as the authors did not calculate the textism ratio—which is a relative measure that takes text length into account—but used raw scores. In addition, textisms at the word level (spelling), rather than at the sentence level, were responsible for this effect.

Other studies looking into children’s grammar abilities did take into account children’s message length when analyzing effects of use of textisms. Kemp et al. [7], Wood et al. [2] and Wood et al. [8] all studied the relationship between children’s texting behavior based on natural messages sent over a 2-day period and their performance on a grammar assessment. Kemp et al. [7] found that primary school children’s (8-10-year old) performance on a grammatical spelling choice task was related to the proportion of grammatical violations they made in their text messages: children who did not perform well on the spelling task made more grammatical violations than children who obtained a better spelling test score. These observations have to do with missing and unconventional punctuation, missing capitalisation, word and grammatical errors (missing words, lack of verbal agreement, verb and preposition merged; and grammatical homonyms). No textisms at the word level were included in this measure. Wood et al. [8], on the other hand, did not find any significant correlations between children’s grammar scores and grammatical violations in their textese. In a longitudinal follow-up study, Wood et al. [2] repeated the procedure used by Wood et al. [8] over a one-year period and again asked children to transcribe their natural messages and assessed their grammar skills on various tasks. For the primary school children, grammatical violations in their text messages did not predict development of their grammatical skills over the year.

In sum, previous studies do not convincingly indicate that use of textese by children negatively affects their conventional writing and spelling abilities. If any association does exist between textism use and literacy, it appears to be positive. Researchers suggest different reasons for this positive association. One of the reasons is that writing text messages is fun and encourages children to play with language without having to worry about spelling conventions. This, in turn, might positively affect children’s attitudes towards other activities associated with literacy (e.g. [12,15,19]). Another important potential advantage of texting is that it increases children’s exposure to text (e.g. [12,17]), which in turn is related to better reading skills [20]; but see [11]. Furthermore, as many textisms are phonologically based (such as ‘2night’ instead of ‘tonight), use of textese is often linked to phonological or phonetic awareness [12,17], which is associated with reading attainment (e.g. [21]). Finally, use of textese could have a more general effect. Previous studies have shown that children know that textisms are not appropriate in, for example, school work. Hence, they are aware of the different registers they can employ. According to Craig [1], this strengthens children’s metalinguistic awareness.

Regarding the effect of children’s use of textese on their grammar abilities the findings are less clear. Two out of the four studies investigating this question found a negative association, whereas two other studies did not obtain any significant correlations. Importantly, the focus of all four studies was on tasks assessing children’s grammar knowledge in written language. Two of the four studies also included a receptive vocabulary task which we will turn to in the discussion section. Written language is considered more formal than spoken language—at least at school and in test situations—which may make children more aware of the necessity to apply conventional (orthographic) grammar rules. In addition, written language, rather than spoken language, allows children time to reread their message, think about its structure consciously and correct it if necessary. As a result, effects of textese on children’s grammar might not be reflected in these type of tasks. Given that textese has properties of both written as well as spoken language (e.g. [12,22]) and spoken language is less bound by formal grammar rules than written language, it is likely that effects of textese on children’s grammar might rather be reflected in tasks assessing children’s grammatical competence in spoken language.

Furthermore, from a linguistic perspective, grammar should be teased apart from orthographic rules, as the latter is not assumed to be part of the core grammatical competence, i.e. the rules that underlie word and sentence formation (morphology and syntax). However, previous studies have used a rather broad definition of grammar when identifying violations in textese and when assessing children’s grammatical competence, which is based on more general written conventions, including omission of capitals and interpunction, for example. As a result, the outcomes of these studies provide limited insight into the effects of textese on children’s grammar proficiency. Therefore, the main aim of the present study is to investigate the effect of textese on children’s language abilities, and specifically their grammar skills.

### Textese and executive control

The secondary aim of this study is to explore the effects of use of textese on children’s cognitive development. Apart from the effects of textese on children’s language skills, it is largely unknown whether and how use of textese influences children’s cognitive development. Research on media multitasking has suggested that frequent switching between different media types—such as typing messages on a phone and watching television simultaneously—and frequent switching between media and non-media activities may negatively impact executive functions, either in daily-life or measured by specific tasks [23] (see [9] for an overview). However, these studies have investigated switching between various types of media and have not focused specifically on mobile phone usage (but see [24]). Hence, it remains unknown whether the use of textese in particular leads to positive or negative effects on children’s executive functions. One reason as to why the use of textese may have a positive effect on children’s executive functioning could have to do with the fact that children who use textese simultaneously activate two registers: textese and conventional written language. The latter is used in more formal situations, such as at school and the former is associated with informal interaction between peers. In some ways, children using these two registers are faced with the same task as bilingual children, who speak two languages. As a result these children, similarly to their bilingual peers, may develop advantages in executive functioning, as will be explained below.

Previous studies with various language populations have found a bilingual advantage on tasks measuring components of executive control (see [10] for a meta-analysis of studies on cognitive correlates of bilingualism). A popular explanation for this bilingual advantage stems from bilinguals’ supposed superior inhibitory control abilities. According to this theory, both languages of bilinguals are simultaneously active (see [25,26] for empirical evidence), which results in competition that makes it necessary to suppress the non-target language when using the other. Due to this simultaneous activation, cognitive, and in particular inhibitory control is trained influencing bilinguals’ behavior in nonlinguistic situations as well. Hence, bilingualism strengthens individuals’ abilities to suppress irrelevant information in situations that require this. Other scholars have argued that it is not inhibitory control in particular that is responsible for the bilingual advantage on executive control, but bilinguals’ superior conflict monitoring system. According to this account, simultaneous activation of languages leads to a conflict, which needs to be resolved by deploying attention-resources. This in turn leads to a better conflict monitoring system in bilinguals (see [27–29] for a review).

As mentioned above, children who are proficient and frequent users of textese may share characteristics with the bilingual populations studied previously with regard to executive functioning advantages—regardless of modality. First of all, children using textese have at least two registers available: textese and a more formal register of conventional writing suitable for school. Secondly, children have to decide when which register is appropriate, in other words, they have to switch between registers. Thirdly, self-reports suggest that both registers are in competition. For instance, participants in a study by Drouin and Davis [30] indicated that textese was hindering their ability to remember standard English spelling. Hence, similarly to bilinguals, proficient texters may have two simultaneously active registers while typing or writing. In order to solve the conflict between the two registers the conflict monitoring system is involved to either suppress the non-target register or select the target variety. This in turn, might improve general cognitive control resulting in better task performance for various executive functions.

To our knowledge, it has not been investigated whether the frequent use of two written registers, instead of two languages, may lead to enhancement of executive control. It has been attested, though, that other skills apart from bilingualism show a positive connection with cognitive abilities, such as playing video games (see [31] for a review) and playing music (e.g. [32,33]). Therefore, a second aim of the present study is to investigate whether proficient texters have a cognitive advantage over non-proficient texters.

### The present study

The main aim of the present study is to investigate whether textese influences children’s grammatical performance in spoken language. It may be expected that using more textese has a positive impact on children’s grammar. The reason is two-fold. First, frequent use of textese may lead to overall better metalinguistic knowledge and a heightened sensitivity to language in general, affecting children’s grammar performance, amongst other aspects of language. If this is indeed so, we would expect a positive relation between various measures of children’s language abilities and a general measure of children’s textese skills, namely textism ratio. This encompasses all modifications at the word level (e.g. clipping of the first or final letter of a word, such as *goin* for *going*; initialisms, such as *lol* for laughing out loud; and number homophones, such as *w8* for *wait*), use of emoticons, and exaggerated use of capitalization and interpunction (e.g. *WHY*???*)*.

Second, the use of textese may have a specific impact on grammar. It is known that other more or less informal written registers allow the omission of words, but do so within the boundaries of the grammar and syntactic rules of the target language. For instance, subjects are usually not left out in English. However, in the so-called diary register subjects of sentences may be dropped as long as this is licensed by the structure of the sentence and its role in the discourse, as is illustrated by a sentence from Fielding’s Bridget Jones’s Diary in (1) (e.g. [34–36]).

(1) Start to wonder whether am really good friend. (p. 261; as cited by Haegeman & Ihsane [36], p. 261)

In headlines, tense marking is omitted and articles are left out [37–39], but the word order rules are still obeyed, and the same holds for sentences used in informal spoken language known as root infinitives [40], such as in so-called Mad Magazin sentences (2).

(2) Him wear a tuxedo?! ([41], p. 2)

In order to decide which element can be or is omitted in each of these registers, the language speaker or writer makes active use of her or his grammar, which may lead to implicit training of the grammar. Given this, we would expect a positive relation between measures of children’s grammar performance and the amount of words they omit in their text messages.

As opposed to the previous line of reasoning we may expect that using more textese has a negative effect on children’s use of grammar in spoken language. Exposure to textese implies exposure to incomplete written sentences. This in turn makes omissions in other areas of language more acceptable, even if they are not. According to this perspective, children are unable to fully separate different registers. Hence, opposite to our previous two hypotheses, this explanation would predict a negative relationship between measures of children’s grammar performance and the amount of words they omit in their text messages.

The secondary aim of this study is to investigate whether textese influences children’s cognitive development. Previous studies with bilingual children have shown that the more proficient a child is in both languages the better some of her or his executive functions (e.g. [42], for working memory; and [43], for inhibition and shifting). We hypothesize that proficient texters share properties with bilingual children. As bilingual children are shown to have better developed executive functions than same-aged monolinguals, we expect proficient texters—as measured by the amount of textisms they use and the words they omit in their text messages—to perform better on tasks measuring executive functions than less-skilled texters.

## Method

### Ethics statement

This study was approved by the Ethics Committee of the Faculty of Social and Behavioural Sciences of Utrecht University (FETC14-001), and there were no objections to the execution of the research project. Parents of each participating child provided written informed consent.

### Participants

Participants were recruited from grade 5 and 6 in six primary schools located in the Netherlands. From the original sample with 67 children, some children were excluded because they were bilingual (N = 4), had a diagnosis of attention deficit disorder (N = 2) or autism (N = 3), or had no experience using WhatsApp (N = 3). This resulted in a final sample of 55 Dutch children, including 28 boys and 27 girls aged 10–13 years (*M* = 10;5, *SD* = 0;8). Children’s nonverbal intelligence was measured using the short version of the Wechsler Nonverbal intelligence scale [44]. This was done to ensure that any relation found between textism use and linguistic or cognitive measures could not be explained by children’s nonverbal intelligence.

Telephone interviews were conducted with one of the parents in order to obtain background information on the child’s development and home environment. Seven children included in the sample had a diagnosis of developmental dyslexia. Additionally, information on parental level of education was gathered. This is the most commonly used measure of socio-economic status (SES) in child research [45]. Parental level of education was calculated as the mean level of education of both parents on a scale from 1 (no education) to 9 (university education). Children’s age, non-verbal intelligence, and SES are presented in Table 1.

**Table 1:** Descriptive statistics for children’s age, raw and norm scores on nonverbal intelligence (IQ) and socio-economic status (SES).

	Age	Nonverbal IQ		SES
		Raw	Norm	
**Mean**	10;5	39.4	106.5	7.2
**St. dev.**	0;8	5.03	12.6	1.5
**Range**	10–13	30–52	85–139	2.5–9

*Note*. Nonverbal IQ scores are based on the short version of Wechsler’s nonverbal intelligence test; SES scores are based on parental education, as gathered through telephone interviews

### Measures and procedures

Participants were individually tested in a quiet room within their own primary school. Testing consisted of one test session that lasted approximately 90 minutes. If a session continued until after school hours, a second session was planned. This was the case for 18 children. Afterwards, children were rewarded for participation with a small present. All computer-administered tasks were programmed and ran using E-Prime 2.0 software [46,47].

#### Use of textese

To gain insight into children’s textism use, two tasks were developed that required children to write text messages. The first task was to reply to a text message. Children were handed a smartphone (Samsung Galaxy Trend Light) containing a text message that included several Dutch textisms. Children were asked to reply to this text message as if they had received it from a friend. The text message in Dutch, Dutch transcription and English translation of the transcription are in Table 2.

**Table 2:** Text message in Dutch, Dutch transcription and English translation.

Utterance	Dutch text message	Dutch transcription	English translation
**1**	heey hgh	Hoi, hoe gaat het?	Hi, how are you?
**2**	wr ben je	Waar ben je?	Where are you?
**3**	vanav afspreke?	Wil je vanavond afspreken?	Do you want to meet tonight?
**4**	Wat wil je doen ☺	Wat wil je doen?	What would you like to do?

In the second task, text messages were elicited through the use of everyday life scenarios (e.g. [16,17,19,48]). In the present study, scenarios were displayed on a computer, including a picture and an auditorily presented story (S1 Appendix). Children were instructed to write a text message in response, again as if they were texting a friend. The task included separate versions for girls and boys. This was done because the stories contained Dutch pronouns, which are marked for gender. Additionally, stories referred to the child’s friend, which is also marked for gender in Dutch (i.e. *vriend* (male friend) and *vriendin* (female friend)). Scenarios were constructed in such a way that children were implicitly instructed to respond with two or three utterances. Each scenario contained several elements that would prompt children to either explain something to his or her friend, and/or to ask a question. Stories were controlled for syllable length and the number of words that could be expressed as textisms (e.g. *school*, *maybe*, *wait*, *tonight* in the examples). Sample scenarios are presented in S1 Appendix.

A total of 10 scenarios were constructed, of which 2 were practice items and 8 were test items. In the practice phase, children were guided (if necessary) to respond to all elements in the scenarios. Such an element could be a sentence that elicits a question or explanation. In the test phase, the experimenter left the room to make the test situation feel more similar to children’s normal ‘texting environment’–without an adult watching them.

In addition to the experimental tasks, we collected text messages from children produced in a more naturalistic setting. First, children (with parental consent) were asked to copy three text messages they had sent from their telephone. Additionally, these children were included in a Whatsapp chat group with their classmates that was monitored by the researchers.

Texting variables were analyzed in the elicited text messages (i.e. collected from the elicited reply and scenarios), the spontaneous messages and the chat groups. This analysis included the calculation of the total number of words and the number of utterances per text message. Subsequently, the number of textisms, omitted words, typos and spelling errors were computed. These were then converted into ratio measures to control for differences in length of the text messages. Textisms included the omission of letters either at the beginning or end of a word (clippings or shortenings), within a word (contractions), unconventional spelling (neologisms), letter/number homophones, the use of English words, slang, orthographic forms of spoken Dutch (accent stylizations), and abbreviations. Relevant for further analyses are textism ratio and omission ratio, which represent use of textese.

#### Texting-related behaviors

Information regarding texting-related behaviors was collected with a questionnaire. This questionnaire was administered at the beginning of the test session and included the following questions: a) whether the child owned her or his own phone, b) how long they had owned the phone, c) the type of telephone used, d) whether they used predictive texting, e) the medium used for texting, f) the frequency of texting, and g) textism use.

The frequency measure is based on children’s average self-reported number of messages sent on a weekday and a weekend day. Time owning a phone (ToP) and importance phone (IP) are based on scale scores. The lowest scale (1) of time owning phone is less than 6 months and the highest scale is longer than 2 years (4). The scale for importance phone ranges from unimportant (1) to very important (5).

#### Language measures

The present study included two standardized Dutch language measures. The Peabody Picture Vocabulary Test (PPVT-3-NL; [49]) is a test of receptive vocabulary. In this task, the child has to choose which one of four pictures corresponds to the word said by the experimenter. The children also completed a sentence repetition task (subtest of the CELF-4-NL; [50]), in which they had to repeat sentences of increasing complexity. Children could make a wide variety of errors in this task, including omission and substitution of words or they could even change the complete syntactic structure. It has been shown that, in order to repeat utterances longer than one’s word span (i.e. the maximum number of random words repeated), individuals must use their syntactic knowledge (e.g. [51,52]). Potter and Lombardi [53] have suggested that participants reconstruct the sentences from lexical, conceptual and syntactic knowledge in long-term memory. In a recent study, Polišenská, Chiat and Roy [54] manipulated sentences in a sentence repetition task with 4 and 5 year old children on various linguistic levels. It turned out that manipulation of the grammatical well-formedness of sentences in particular, i.e. word order and substitution of function words, impacted children’s ability to repeat a sentence correctly, and highlighted the influence of morphosyntactic knowledge on these type of tasks. The general consensus is that sentence repetition tasks reflect general language abilities and syntactic competence, rather than assessing children’s working memory skills (e.g. [55,56]).

#### Executive functions

For the purpose of this research, we selected tasks that have been used in previous research with bilingual children and that showed bilingual advantage. These comprise attention and inhibition tasks in which children have to ignore distracting and interfering information [28,57,58], verbal working memory tasks [42,59] and visuospatial working memory tasks [60].

Selective attention (i.e. the ability to focus on relevant stimuli, while filtering out irrelevant information) was measured through the subtest ‘Sky Search’, subpart of the Test of Everyday Attention for Children (TEA-Ch; [61]). Children were presented with an A3 paper with 64 space ship pairs. They were instructed to circle identical space ship pairs (target) as quickly as possible while ignoring non-identical pairs of space ships (distractor). Participants also completed a motor-control version of the task in which only the identical space ship pairs were presented. The scores on the Sky Search task were computed by dividing the time necessary for a child to complete the test phase and the motor control phase by the number of correctly identified pairs of spaceships for each phase separately. Subsequently, the outcome for the motor control phase was subtracted from the score for the test phase. Thus, motor speed was controlled for in the calculation of the selective attention score.

Visuo-spatial working memory scores were obtained through the subtest ‘Spatial orientation’ of the Wechsler Nonverbal intelligence scale [44]. The experimenter sat opposite the participant with a fixed arrangement of blocks between them. The experimenter would then point to these blocks in sequences consisting of 2 to 9 items. Children were asked to repeat the sequence in a backward condition. Each level consisted of two sequences and children had to answer at least one item correctly in order to move on to the next level. The score reported is the total number of sequences the child could correctly repeat (a maximum of 16).

Verbal working memory was tested using a computerized version of the backward digit span based on the Automated Working Memory Assessment (AWMA; [62]). In this test, children would see Bugs Bunny on the computer screen and hear sequences of 1–8 digits. Children were instructed to repeat the sequence backwards. Children had to correctly repeat 4 trials (out of a maximum of 6) to proceed to the next level. The scores reported in the present study were calculated following the AWMA conventions: for each block that children completed, they were awarded at least 4 points. When a child had a score of 4 out of 4, 6 points were assigned. When children scored of 4 out of 5, they were awarded 5 points. When children failed to correctly backwardly repeat 4 items within a block, they did not receive any points in that block. The total score was the sum of the scores per block.

For both memory tasks (visuo-spatial and verbal) a forward condition was completed as well. Previous research has indicated that visuospatial short-term memory tasks draw on executive control, and do so more than verbal short-term memory tasks [42,63]. For this reason we decided to include the forward condition of the Spatial orientation task in analyses of children’s executive functioning by calculating one visuo-spatial memory score. Verbal short-term memory was used as a control variable in the analyses when relevant, but was not included in the verbal working memory score.

Interference inhibition was measured with a Flanker task, administered on a computer (modified from Rueda et al. [64], see [57]). The test consisted of trials containing five fish in a horizontal row presented on the computer screen. Children were instructed to indicate the direction of the middle fish by pressing on the left or right response buttons on each side of the keyboard. The task consisted of 2 blocks of 20 test trials, preceded by 8 practice trials. Each trial was followed by feedback. 50% of the trials were congruent (all fish point in the same direction); the other 50% were incongruent (the direction of the middle fish deviates from the others). Reaction times and accuracy of each response were recorded by E-prime. Processing the data, inaccurate trials, reaction times that deviated 3 standard deviations from the mean, and reaction times below 200 ms were excluded from analysis. These criteria resulted in the removal of 62 trials (3.2%). Results from 5 children were missing due to technical problems. One child had an average reaction time more than 3 standard deviations above the group average on the global reaction times, congruent trials and incongruent trials, and his results on the Flanker task were removed from the dataset. Subsequently, means for congruent and incongruent trials and a difference score (i.e. the Flanker effect) were calculated per child.

## Results

In this section we will first compare children’s use of textese in the various types of messages collected. Then we will present the general descriptions of children’s texting behavior, followed by the outcomes of analyses relevant to the main research question addressing the relation between use of textese and language skills. In the final part of this section results related to the second research question, on the relation between texting proficiency and executive functions, are presented.

### Use of textese in different type of messages

Table 3 presents the mean textism and omission ratio for the two types of elicited text messages, and children’s spontaneous messages. Since only 12 children participated in the chat groups, these results are not displayed. Use of textisms was highest in the spontaneous messages and more or less similar between both elicitation tasks. Omission of words was highest in the elicited reply task and lower for the scenarios and the spontaneous messages. A Friedman test was conducted for both ratios separately to see whether type of message (spontaneous messages, elicited reply task, elicitation through scenarios) affected the outcomes. For both textism ratio and omission ratio the effect of type of message was significant (textism ratio: *X*^*2*^(2) = 6.24, *p* = .04; and omission ratio: *X*^*2*^(2) = 26.52; *p* < .01). Post hoc analyses with Wilcoxon signed-rank tests were conducted with a Bonferroni correction, resulting in a significance level set at *p* < .017. Textism ratio was higher in the spontaneous messages than in children’s elicited replies (*Z* = -2.55, *p* = .01), while the omission ratio was higher in elicited replies than in children’s spontaneous messages (*Z* = -4.63; *p* < .01). The omission ratio in the elicited replies was also higher than in the task with scenarios (*Z* = -5.57, *p* = < .01). All other differences in textism or omission ratio between types of messages were not significant.

**Table 3:** Means, standard deviations and ranges of children’s use of textese in various types of messages.

	Elicited reply		Scenarios		Spontaneous messages	
	Textism ratio	Omission ratio	Textism ratio	Omission ratio	Textism ratio	Omission ratio
**Mean**	13.50	27.88	13.62	9.21	24.16	7.37
**St. dev.**	15.67	17.59	11.75	7.49	28.92	8.22
**Range**	0–53.85	0–63.64	0–51.50	0–37.84	0–150.00	0–25.00
**N**	55	55	55	55	35	35

Table 4 shows the correlations between the three types of text messages. For textism ratio significant and moderate correlations were found between the elicited reply and the scenarios, and between the scenarios and the spontaneous messages. The relation between the elicited replies and the spontaneous messages was marginally significant. These findings are similar to findings from Plester, Lerkkanen et al. [16] and suggest that children’s personal texting style was maintained in the elicited messages. There were no significant correlations for omission ratio. In the next analyses only children’s textism and omission ratio on the elicitation tasks were used, because data on these tasks was available for all children.

**Table 4:** Correlation matrix of children’s textism ratio and omission ratio on the elicitation tasks and in their spontaneous messages.

	Textism ratio			Omission ratio		
	Elicited reply	Scenarios	Spontaneous messages	Elicited reply	Scenarios	Spontaneous messages
**Elicited reply**	1.00	.59**	.29^†^	1.00	.20	.17
**Scenario’s**	55	1.00	.54**	55	1.00	.08
**Spontaneous messages**	35	35	1.00	35	35	1.00

^†^*p* < .1;

*p < .05;

**p < .01

### Texting variables

In Table 5 the average numbers and ratios are presented for various texting variables. Words and utterances are based on the average number per child. For frequency the median is displayed as some children reported extreme values that strongly influenced the mean.

**Table 5:** Means, standard deviations and ranges of texting characteristics.

	Words	Utterances	Textism ratio	Omission ratio	Typo ratio	Error ratio	Frequency	ToP	IP
**Mean**	101.82	21.51	12.69	13.26	2.78	2.50	26.00	2.33	3.79
**St. dev.**	29.72	4.30	10.54	9.75	3.62	1.87	104.19	1.16	0.91
**Range**	48–175	14–33	0–50.0	1.4–54.2	0–18.2	0–7.6	0–700	0–4	2–5

*Note*. ToP = time owning phone; and IP = importance phone.

Slightly less than 13% of the total amount of the text messages were written as textisms and little over 13% of the total amount of the words in text messages were omitted. Children’s texting behaviour varied considerably: some children did not use textisms at all and omitted very few words, whereas other children substituted 50% of conventional words by textisms and omitted more than 50% of the words (see S2 Appendix for an overview of the various types of textisms used). Few typos and spelling errors were made (less than 3% of the words). Whereas the majority of children reported sending between 0 and 45 text messages on average per day (*N* = 46), some children reported sending over 100 text messages (*N* = 5) and one child reported sending 800 text messages on an average day during the weekend. These outliers might have strongly influenced the outcomes of further statistical analyses, and therefore, the extreme values were excluded from the dataset. These comprised values above 100 messages sent per day on average for texting frequency (*n* = 5); and values with a z-score above 3.0 for textism ratio (*n* = 2) and omission ratio (*n* = 2).

### Textese and language skills

We hypothesized that textese may have an effect on children’s language skills in general, possibly affecting both vocabulary and their grammar. In addition to vocabulary and grammar scores, nonverbal IQ and verbal short-term memory are presented in Table 6, as these are considered indicators of language aptitude (e.g. [65–67]) and therefore potentially correlate with the language outcomes of the present study. Raw scores are used in further analyses.

**Table 6:** Children’s mean raw and norm scores, standard deviations and ranges on the receptive vocabulary, grammar, nonverbal intelligence and verbal short-term memory task.

	Vocabulary		Grammar		Nonverbal IQ		VSTM
	Raw	Norm	Raw	Norm	Raw	Norm	Raw
**Mean scores**	132.7	105.2	67.51	9.9	39.4	106.5	27.0
**St. dev.**	9.79	10.85	9.98	2.24	5.03	12.59	3.54
**Range**	103–150	72–135	43–85	5–14	30–52	85–139	19–36

*Note*. VSTM = verbal short-term memory.

Correlations between the texting variables—texting frequency, textism ratio and omission ratio—the two language scores and the background variables age, SES, nonverbal IQ and digit span are presented in the upper right triangle of the correlation matrix in Table 7 and the number of participants on which the correlations were based in the lower left triangle. Children’s age, SES, nonverbal IQ and texting frequency were not significantly related to children’s vocabulary and grammar scores. Children’s digit span was marginally significantly and positively correlated with vocabulary skills and significantly and positively correlated with grammar abilities. Furthermore, there was a significant positive relationship between age and textism ratio and age an omission ratio; and a marginally positive significant relationship between texting frequency and textism ratio. Textism ratio and omission ratio were both positively and (marginally) significantly correlated with children’s vocabulary and grammar scores. The relation between textism ratio and omission ratio was not significant, which offered support for treating these two ratios as separate components of textese. Finally, children’s scores on the vocabulary and grammar tasks correlated positively.

**Table 7:** Correlation matrix of children’s background measures, use of textese, frequency of texting and performance on the language tasks.

	Age	SES	Nonverbal IQ	VSTM	Frequency	Textism ratio	Omission ratio	Vocabulary	Grammar
**Age**	1.00	-.39**	-.02	-.03	-.02	.31*	.27*	.10	.02
**SES**	55	1.00	.17	-.01	-.20	-.23	-.16	-.06	-.04
**Nonverbal IQ**	55	55	1.00	.26^†^	-.03	-.06	.02	.21	.16
**VSTM**	55	55	55	1.00	.25^†^	.20	.16	.24^†^	.44**
**Frequency**	50	50	50	50	1.00	.28^†^	-.15	.05	-.02
**Textism ratio**	53	53	53	53	48	1.00	.12	.27*	.33*
**Omission ratio**	53	53	53	53	48	51	1.00	.21^†^	.32*
**Vocabulary**	55	55	55	55	55	53	53	1.00	.61**
**Grammar**	55	55	55	55	55	55	55	55	1.00

*Note*. SES = socioeconomic status; and VSTM = verbal short-term memory.

^†^*p* < .1;

*p < .05;

**p < .01

To answer our research question, two hierarchical regression analyses were conducted with respectively vocabulary and grammar performance as the dependent variable. Age was included as control variable in the first step of both analyses. Forward digit span was added because it correlated with both vocabulary and grammar scores. Textism ratio and omission ratio were included in the second and third step respectively. In the final step of the analyses with vocabulary score as dependent variable, grammar performance was added, and vice versa in the analysis with grammar score as dependent variable. This was done because vocabulary and grammar were correlated with each other.

#### Vocabulary

In the first step, digit span, but not age, was a significant predictor of children’s vocabulary scores, as presented in Table 8. As the returned standardized residuals for the full sample were not normally distributed due to one low vocabulary score (z = -3.03), the analysis presented here is conducted without the data of this child. Although the predictive strength of the model slightly increased by adding textism ratio in the second step, the variable did not improve the model significantly. The same was true for omission ratio in the third step. Finally, by adding children’s grammar scores as predictor, the model gained in predictive power, *R*^2^ = .46, *F*(5, 44) = 7.56, *p* < .001. The only significant predictor in the final model was grammar scores.

**Table 8:** Hierarchical regression analysis with children’s receptive vocabulary scores as dependent variable.

	ΔR^2^	B	SE B	β
**Step 1**	.14*			
Constant		94.08	23.19	
Age		1.23	1.78	.09
VSTM		0.95	0.35	.36*
**Step 2**	.03			
Constant		104.84	24.45	
Age		0.32	1.90	.02
VSTM		0.87	0.36	.33*
Textism ratio		0.18	0.14	.19
**Step 3**	.04			
Constant		120.79	26.27	
Age		-1.01	2.07	-.08
VSTM		0.70	0.37	.27^†^
Textism ratio		0.18	0.14	.19
Omission ratio		0.51	0.33	.23
**Step 4**	.25**			
Constant		84.72	23.34	
Age		0.79	1.77	.06
VSTM		0.07	0.34	.03
Textism ratio		-0.02	0.12	-.02
Omission ratio		0.09	0.29	.04
Grammar		0.56	0.12	.65**

*Note*. VSTM = verbal short-term memory.

^†^*p* < .1;

*p < .05;

**p < .01

#### Grammar

Similar to the previous model, digit span was a significant predictor of children’s grammar scores in the first step—and remained so throughout the analysis—whereas age was not. By adding textism ratio in the second step and omission ratio in the third step, the model improved significantly; digit span, textism ratio and omission ratio were all three significant predictors. The addition of children’s vocabulary scores in the final model further improved its predictive value, *R*^2^ = .49, *F*(5, 45) = 8.71, *p* < .001. By adding this variable, textism ratio became no longer significant as predictor, whereas digit span and omission ratio remained significant predictors (see Table 9).

**Table 9:** Hierarchical regression analysis with children’s grammar scores as dependent variable.

	ΔR^2^	B	SE B	β
**Step 1**	.25**			
Constant		19.40	25.21	
Age		0.65	1.94	.04
VSTM		1.52	0.38	.50**
**Step 2**	.07*			
Constant		37.84	25.70	
Age		-0.88	1.99	-.06
VSTM		1.36	0.38	.45**
Textism ratio		0.32	0.15	.29*
**Step 3**	.07*			
Constant		59.44	26.53	
Age		-2.60	2.06	-.17
VSTM		1.10	0.38	.36**
Textism ratio		0.29	0.14	.26*
Omission ratio		0.59	0.26	.30*
**Step 4**	.11**			
Constant		13.35	28.57	
Age		-2.18	1.90	-.14
VSTM		0.79	0.36	.26*
Textism ratio		0.19	0.13	.17
Omission ratio		0.50	0.24	.25*
Vocabulary		0.39	0.13	.37**

*Note*. VSTM = verbal short-term memory.

^†^*p* < .1;

*p < .05;

**p < .01

In order to investigate if digit span and omission ratio explained unique variance, two additional analyses were performed in which digit span and omission ratio were entered last in the hierarchical regression analysis. Digit span explained unique variance as indicated by a significant *R*^2^ change after adding children’s digit span scores, *ΔR*^2^ = .06, *ΔF*(1, 45) = 4.93, *p* = .03, and the same held for omission ratio, *ΔR*^2^ = .06, *ΔF*(1, 45) = 5.08, *p* = .03.

### Textese and executive functions

The second research question asked whether skilled texters have better executive functions than less skilled texters. An overview of scores on the four tasks measuring executive functions is given in Tables 10 and 11.

**Table 10:** Mean scores, standard deviations and ranges for children’s selective attention, visuospatial working memory and digit span backwards scores.

	Selective attention	VSM	VWM
**Mean scores**	2.99	16.40	14.60
**St. dev.**	.83	3.11	3.86
**Range**	1.72–5.33	10–24	8–28

*Note*. VSM = visuospatial memory; and VWM = verbal working memory.

**Table 11:** Mean scores, standard deviations and ranges for the children’s reaction time on the Flanker task.

	Global	Congruent trials	Incongruent trials	Flanker effect
**Mean scores**	618.82	601.31	636.87	35.57
**St. dev.**	103.32	103.36	108.51	48.31
**Range**	447.33–846.92	449.25–844.11	404.63	-52.11–197.54

Correlations between the various variables are in the upper right triangular in Table 12. The number of participants included in the correlations is given in the lower right triangular in the same table. A marginally negative significant correlation between textism ratio and selective attention scores indicated that the more textisms children use, the better their selective attention is (as a lower score on this task was indicative for a better performance). A marginally significant correlation between SES and children’s selective attention scores suggested that higher SES is associated with better selective attention. There were no significant positive correlations between any of the texting variables—textism ratio, omission ratio, and texting frequency—and the outcomes on the other executive function tasks.

**Table 12:** Correlation matrix of children’s performance on the executive functions tasks.

	Selective attention	VSM	VWM	Flanker global RTs	Flanker congruent RTs	Flanker incongruent RTs	Flanker effect
**Age**	-.02	.01	.05	.02	.03	.01	-.16
**SES**	.23^†^	.06	.07	.03	.05	-.01	-.04
**Nonverbal IQ**	-.13	.74**	.13	-.38**	-.35*	-.43**	-.07
**VSTM**	-.10	.11	.35**	-.35*	-.40**	-.29*	.15
**Frequency**	-.17	.00	-.07	-.02	-.00	-.02	.05
**Textism ratio**	-.24^†^	-.14	-.26	-.08	-.07	-.07	-.09
**Omission ratio**	-.02	.11	-.05	-.17	-.19	-.14	.11
**Selective attention**	1.00	.05	-.05	.05	.08	.01	-.10
**VSM**	55	1.00	.07	-.34*	-.29*	-.38**	-.09
**VWM**	55	55	1.00	-.12	-.16	-.10	.03
**Flanker global RTs**	49	49	49	1.00	.97**	.97**	.11
**Flanker congruent RTs**	49	49	49	49	1.00	.90**	-.07
**Flanker incongruent RTs**	49	49	49	49	49	1.00	.30*
**Flanker effect**	49	49	49	49	49	49	1.00

*Note*. VSM = visuospatial memory; VWM = verbal working memory; RT = reaction time; SES = socioeconomic status; VSTM = verbal short-term memory.

^†^*p* < .1;

*p < .05;

**p < .01

The relation between textism ratio and children’s selective attention scores was further investigated by means of a hierarchical regression analysis. Age was included as predictor in the first step. As there was some evidence for a relation between parents’ SES and children’s textism ratio and scores on the selective attention task, this variable was included as a control variable in the first step as well. Furthermore, children’s texting frequency was added in the second step, as this variable was indicative of the amount of use of the texting register by the children and therefore an indicator of the degree in which they were trained in managing two orthographic conventions, texting and standard spelling. Adding age and SES in the first step of the analysis and texting frequency in the second step did not improve the model significantly. Textism ratio did not improve the model either (*R*^*2*^ = .10, *ΔR*^*2*^ = .05, *F*(4 41) = 1.09, and *p* = .37).

## Discussion

The main aim of this study was to investigate whether textese influences children’s grammatical performance in spoken language. First of all, we hypothesized that a positive relationship may be found between children’s textism ratio and their performance on the language tasks in general. The reason is that use of textese may lead to an improvement in children’s metalinguistic awareness and heightened sensitivity to language [12,19]. Secondly, we hypothesized that omitting words in textese on a frequent basis would result in additional training of the grammar system. The assumption that underlies this hypothesis is that omission in textese is rule-based similarly to omissions in other informal registers [34–36,41]. A third, alternative, hypothesis is possible as well. Frequent omission of words in text messages may potentially lead to the opposite effect if children are not sufficiently able to separate grammar rules in textese from grammar rules in spoken language. If this holds true, children who frequently omit words in their text messages are more likely to omit words in other registers in which this is not allowed. According to this third hypothesis, there may be a negative relationship between omission ratio in children’s text messages and their performance on the sentence repetition task that was administered to measure children’s grammar performance.

Our results revealed no clear support for the first hypothesis. At first sight, children’s textism ratio correlated positively with their performance on the receptive vocabulary and the grammar tasks. Hence, the more textisms children used in their text messages the higher their vocabulary and grammar scores, as hypothesized. However, in the subsequent regression analyses these patterns were no longer attested, as textism ratio was neither a significant predictor of variance in vocabulary scores nor grammar scores after age, verbal short-term memory, omission ratio and either vocabulary or grammar scores were controlled for. These findings are in accordance with two previous studies by Plester and colleagues [16,17]. In the first study no relationship was found between the British Picture Vocabulary Scales II [68] and children’s textism ratio. In [16] only a marginally significant correlation was obtained between children’s textism ratio and their performance on a shortened Finnish version of the Peabody Picture Vocabulary Test-Revised [69,70].

A clear relationship was found between children’s omissions in their text messages and their grammar performance, as predicted by the second and third hypotheses. Not only was there a significant correlation between the two measures, omission ratio was also found to be a significant predictor of variance in children’s grammar scores when children’s age, verbal short-term memory, vocabulary score, and textism ratio were controlled for. The direction of this relation was positive: the more words children omitted in their text messages, the better their grammar performance. This suggests that omitting words while using textese may lead to training of the grammar system, resulting in improved grammar performance in spoken language.

Given previous, in general positive, results on the influence of textese on children’s literacy development, our results seem to concur (see [5] for an overview). However, in previous research no evidence was found for positive relationships between use of textese and children’s (written) grammar performance. Importantly and in contrast with previous studies, conventions in writing were separated from grammar (i.e. morphological and syntactic rules) in the present study, both in the textese register and in the language measures. One previous study included both orthographic violations as well as morphological and syntactic violations in their measure of structural textisms [7], while a second study [6] did not include morphological and syntactic violations in their textese measure. Furthermore, all studies assess children’s grammar performance on written tasks. As writing is relatively formal compared to spoken language, children may have been highly conscious of the rules of (written) grammar in these tasks.

A few studies used (in addition to tests of written language) also grammar comprehension in spoken language (Task of Receptive Grammar II: TROG II; [71]). However, even when use of textese was measured via the omission of words, no significant relationships emerged with grammar performance ([2,8]). There may be at least two reasons for the discrepancy between the findings in these studies and the present study. First of all, the TROG II score does not only contain grammatical knowledge, but also receptive vocabulary performance, similar to the PPVT used in the present study. In the present study, no relationship was found between children’s outcomes on the receptive vocabulary task and their omission ratio in textese. Thus, the lack of correlations between children’s performance on the TROG II and the amount of words they omit, may be caused by the hybrid nature of the language measure which includes both grammar and vocabulary knowledge. Secondly, the TROG II is a receptive task in which children select the corresponding picture out of a set of four after hearing a sentence or a word; this task may be relatively insensitive compared to the sentence repetition task used in the present study. In a sentence repetition task the sentence is not only interpreted, as in the TROG II, but also reconstructed.

Our study offers support for treating textese as a separate register, as advocated by Bernicot et al. [14], with its own set of rules. By using textese—and as a consequence omitting words in their messages—children apply rules of grammar and do so in a context-sensitive manner. In order to decide which words to drop in which context, children analyse their sentences and in doing so, are constantly training their grammatical knowledge, and strengthening their grammar system. Our results show that use of textese is associated with children’s general grammar performance. A next step would be to find out whether use of textese is related to more specific components of grammar. One way to do so would be to analyse the type of errors on this task in more depth. We leave this for future research. Note furthermore that the relationship between use of textese and grammar performance could also be argued to be reversed or bidirectional (see for example [72] for a discussion on the bidirectional association between textese and literacy). Especially with regard to the relationship found between the amount of omissions in children’s text messages and their grammar performance the effect may go in both directions because children with better grammar competence may be more likely to know which omissions are allowed under which circumstances and which are not. Possibly, when these children just start texting they are more likely to omit words than other new texters. As we also assume that frequently omitting words while texting will lead to additional training of the grammar system, we would expect that this early effect is later on reversed: the more children omit in their text messages, the better their grammar skills. The present study found evidence for this later effect. Longitudinal research is necessary to further investigate this relationship.

Our second research question asked whether skilled texters have better executive functions than less skilled texters. We did not find convincing evidence for the relationship between the texting variables and scores on the various executive function tasks, but the results may suggest some trends in the expected direction. A negative and marginally significant relationship was attested between textism ratio and children’s selective attention scores: children who used more textisms—and can therefore be argued to be more skilled texters—performed better on the selective attention task than other children.

Two implications can be drawn from these findings. First of all, this study did not find clear support for the idea that the bilingual advantage can be generalized to the combination of conventional writing and textese. This does not necessarily entail, though, that being proficient in textese (apart from conventional writing) does not influence children’s executive functions. Previous work with bilingual children has shown that a bilingual advantage is not obtained at all times and can be dependent on bilingual proficiency and length of exposure [42,73,74]. Also, when it is attested, this is mainly with relatively young children or older participants [29]. The children in the present study are already slightly older and may show less variance in executive functions, reducing the chances of obtaining a significant effect.

A second implication that can be drawn from these results is that frequent and skilled texters do not seem to suffer from poorer executive functions due to media multitasking. Previous studies have suggested that frequent simultaneous engagement in two or more types of media or using media when engaged in non-media activities may have negative implications for children’s and adolescents’ executive functions. We hypothesized that children who frequently text may also be more likely to frequently media multitask and therefore be more vulnerable to negative effects on their executive functions. Our study did not support this prediction. This is not entirely surprising, as previous studies mainly attested a negative effect on media switching on participants’ self-reported executive functions in daily life, and less so on executive functions measured by neuropsychological tasks [9,23]. Neither can we rule out the possibility that negative effects on the executive functions of skilled and proficient texters are cancelled out by a positive “bilingualism” effect. This would explain why neither a positive effect, supporting the bilingualism hypothesis, nor a negative effect, supporting the media multitasking hypothesis, was found. Future research with various age groups asking participants about their media switching behaviour and their executive functions in daily life is necessary to further explore the effects of texting on executive functions.

A limitation of the study concerns the way in which text messages were collected. The most natural representation of children’s textese is in their spontaneous messages. However, as attested also by previous studies (e.g. [16,17]) and the present study not all parents (and children) were willing to provide children’s text messages, often due to privacy issues. Limiting the present study to spontaneous messages would have led to a smaller sample size, affecting the power of the study. Instead, we chose to use two types of elicitation methods to gain insight in all children’s texting behaviour. In line with findings form Plester, Lerkkanen et al. [16], the task using scenarios elicited fewer textisms compared to the task asking children to reply to a text message and children’s spontaneous text messages. The same was true for the amount of omissions in children’s text messages. Still, correlations between the elicited and natural message types showed that children’s response on the scenarios reflect their natural use of textisms. For the elicited replies this was only weakly so. These patterns are similar to findings from Plester, Lerkkanen et al. [16]. For omission ratio, no clear relationship was obtained between the spontaneous messages and the elicited messages. This may have been due to the instructions for copying spontaneous messages, as we asked for longer messages, which probably have fewer omissions than shorter messages. Ideally in future research, experimenters could make use of interventions ([11,14]). Mobile phones could be distributed to children having no experience with texting yet. In return, children should be asked to provide the researchers their text messages on a regular basis. Their performance on various tasks measuring language skills and executive functions could be compared to their texting behaviour over time and a control group of children not owning a phone. This method would make it possible to gain access to children’s natural text messages and would, at the same time, offer further insight into the direction of causal relationships.

## Conclusions

The present study has extended the existing body of knowledge about the effects of textese on written language by investigating the influence of textese on children’s grammar performance in spoken language. Not only may textese improve children’s abilities in written language, as has been attested in previous work, it may also enhance their grammar abilities in spoken language as the present study has shown. This clearly refutes the suggestion that use of textese may lead to language deterioration. Furthermore, textese did not influence children’s performance on various tasks measuring executive functions, regardless of the potentially negative effects of media multitasking—something that may be stimulated by mobile phone use—on executive functions. Hence, this study did not find any evidence of negative influences of textese on children’s grammar performance or executive functions. On the contrary, if an effect was found, it was positive, indicating that children benefit from texting.

## Supporting Information

S1 Appendix Sample scenarios. (DOCX)

S2 Appendix Type of textisms used by children in their elicited text messages. (DOCX)
