# Supplementary material for: The Influence of Texting Language on Grammar and Executive Functions in Primary School Children
Source: PLoS One. 2016 Mar 31;11(3):e0152409. doi: 10.1371/journal.pone.0152409 (PMC4816572; doi:10.1371/journal.pone.0152409)
Supplement: S1 Appendix — (DOCX) [file pone.0152409.s001.docx]

**S1 Appendix – Sample scenarios**

| 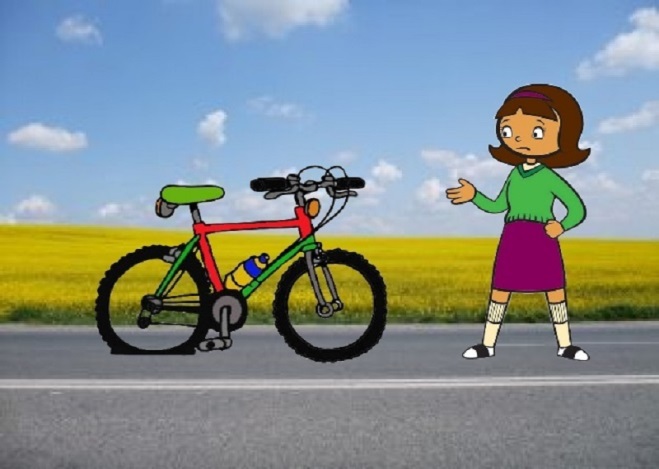 | 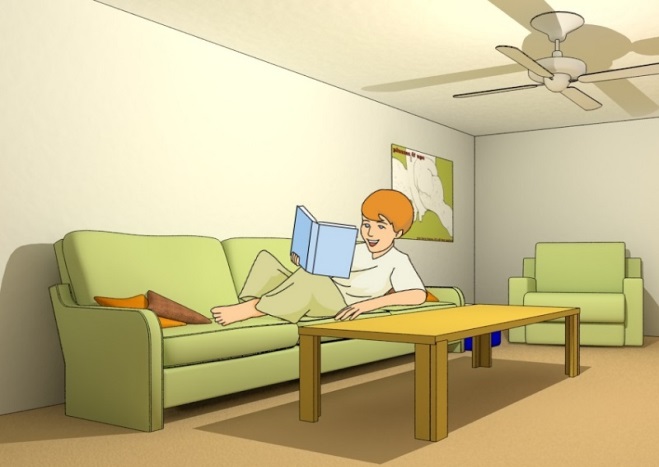 |
| --- | --- |
| *(a)*  You are on your way to a friend to bike to school together, but you get a flat tire.  You explain to her what has happened.  You ask her if she can maybe wait for you. | *(b)*  You’re at home tonight and are bored.  You ask a friend what he’s doing.  You ask whether he maybe wants to meet tonight to play computer games. |
