# Supplementary material for: The Influence of Texting Language on Grammar and Executive Functions in Primary School Children
Source: PLoS One. 2016 Mar 31;11(3):e0152409. doi: 10.1371/journal.pone.0152409 (PMC4816572; doi:10.1371/journal.pone.0152409)
Supplement: S2 Appendix — (DOCX) [file pone.0152409.s002.docx]

**S2 Appendix – Type of textisms used by children in their elicited text messages**

| **Category** | **Definition and examples** | **Total number of occurrences** |
| --- | --- | --- |
| Clippings | Omission of the first or final letter of a word – *lope* (lopen; *to walk*), *k* (ik; *I*) | 110 |
| Contractions | Omission of letters within a word – *srry* (sorry), *wrm* (waarom; *why)* | 88 |
| Shortenings | Omission of the last letters of a word – *miss* (misschien; *maybe*), *vanav* (vanavond; *tonight*) | 40 |
| Neologisms | Word written differently from conventional spelling – *nii* (niet; *not*), *leuwk* (leuk; *nice*) | 159 |
| Letter/number homophones | Similar sounding letter or number replacing a letter combination – *w8* (wacht; *wait*), *suc6* (succes; *success*) | 16 |
| Onomatopoeias | *haha*, *oeps* (*oops*) | 2 |
| English words | English words within Dutch text, irrespective of spelling – *plzzz*, *tnx* | 26 |
| Slang | Informal by young people spoken language variety – *yoo* (*yo*); *jo gast* (*yo dude*) | 19 |
| Accent stylizations | Orthographic representation of spoken Dutch – *morgu* (morgen; *tomorrow*), *das* (dat is; *that is*) | 19 |
| Initialisms and abbreviations | Words represented by their initial letter and unconventional abbreviated words – *hgh* (hoe gaat het; *how are you*), *ff* (even; *for a while*) | 74 |
| Emoticons | ☺, ☹ | 79 |
| Informal interpunction and capitalization | Informal use of interpunction and capitalization - *!!!*, *HOI* (*hi*) | 60 |
| **Total** | | **692** |

Interpunction and capitals were omitted in 87.9% of the obligatory contexts with little variation between children. Therefore, these occurrences were not counted as textisms and not included in the table.
